# Confirmation of a Two-Factor Solution to the Questionnaire of Cognitive and Affective Empathy in a French Population of Patients With Schizophrenia Spectrum Disorders

**DOI:** 10.3389/fpsyt.2019.00751

**Published:** 2019-10-25

**Authors:** Eric Brunet-Gouet, Nils Myszkowski, Mickael Ehrminger, Mathieu Urbach, Bruno Aouizerate, Lore Brunel, Delphine Capdevielle, Isabelle Chereau, Caroline Dubertret, Julien Dubreucq, Guillaume Fond, Christophe Lançon, Sylvain Leignier, Jasmina Mallet, David Misdrahi, Sylvie Pires, Priscille Schneider, Franck Schurhoff, Hanan Yazbek, Anna Zinetti-Bertschy, Nadine Bazin, Christine Passerieux, Franck Zenasni, Paul Roux

**Affiliations:** ^1^FondaMental Foundation, Créteil, France; ^2^Department of Adult Psychiatry, Versailles Hospital, Le Chesnay, France; ^3^HandiRESP Laboratory, EA4047, Health Sciences Department Simone Veil, University of Versailles Saint-Quentin-En-Yvelines, Montigny-le-Bretonneux, France; ^4^Department of Psychology, Pace University, New York, NY, United States; ^5^Laboratoire Adaptations Travail-Individu, Université Paris Descartes, Université Sorbonne Paris Cité, Boulogne-Billancourt, France; ^6^Department of Adult Psychiatry, Charles Perrens Hospital, F-33076 Bordeaux, France; Laboratory of Nutrition and Integrative Neurobiology (UMR INRA 1286), University of Bordeaux, France; ^7^INSERM U955, Translational Psychiatry Team; AP-HP Mondor University Hospital, DHU Pe-PSY, Schizophrenia Expert Center, Creteil, France; ^8^University Department of Adult Psychiatry, Hospital La Colombière, CHU Montpellier, France; INSERM, Univ Montpellier, Neuropsychiatry: Epidemiological and Clinical Research, Montpellier, France; University of Montpellier, Montpellier, France; ^9^CHU Clermont-Ferrand, Service de Psychiatrie B, Université Clermont Auvergne, Clermont-Ferrand, France; ^10^AP-HP; Department of Psychiatry, Louis Mourier Hospital, Colombes, France; Inserm UMR1266, Institute of Psychiatry and Neuroscience of Paris, University Paris Descartes, France; Université Paris Diderot, Sorbonne Paris Cité, Faculté de Médecine, France; ^11^Psychosocial Rehabilitation Reference Centre, Alpes Isère Hospital, Grenoble, France; ^12^La Conception Hospital, AP-HM, Aix-Marseille Univ, School of Medicine - La Timone Medical Campus, EA 3279: CEReSS - Health Service Research and Quality of Life Center, Marseille, France; ^13^Ste Marguerite Hospital, AP-HM, Aix-Marseille Univ, School of Medicine - La Timone Medical Campus, EA 3279: CEReSS - Health Service Research and Quality of Life Center, Marseille, France; ^14^Department of Adult Psychiatry, Charles Perrens Hospital, F-33076 Bordeaux; University of Bordeaux, CNRS UMR 5287-INCIA, Bordeaux, France; ^15^University Hospital of Strasbourg, Department of Psychiatry, Strasbourg, France; University of Strasbourg, Strasbourg, France; Inserm U1114, Strasbourg, France

**Keywords:** schizophrenia spectrum, empathy, assessment method, quality of life, functioning

## Abstract

The Questionnaire of Cognitive and Affective Empathy (QCAE) is a tool for self-assessing the cognitive and emotional components of empathy. A study showed that a two-factor model fits the data of patients with schizophrenia, whereas other reports on healthy subjects have suggested a five-factor decomposition. We aimed to replicate the model of *Horan* et al. in a French population with schizophrenia spectrum disorders (i.e., schizophrenia and schizoaffective disorders) participating in the EVACO Study (NCT02901015). In total, 133 patients were assessed with the QCAE, the *Positive and Negative Symptom Scale* (PANSS), the *Personal and Social Performance Scale* (PSP), and the *Self rating Quality of Life Scale* (S-QoL). The two-factor model demonstrated an adequate fit with the data, comparable to that reported by Horan *et al*. Males scored higher on the Affective subscore than females. After correction for multiple tests, psychopathology (PANSS) and functioning (PSP) did not correlate significantly with the QCAE subscores. However, quality of life (S-QoL) correlated positively with the Emotional Contagion subscore. Thus, the variability of empathetic disposition in schizophrenia may be considered through the cognitive *versus* affective dichotomy and properly investigated with the QCAE. The results support further investigation of the relationship between QCAE scores and subjective outcome measurements, such as quality of life, and emphasize the importance of cross-cultural comparisons.

## Introduction

Schizophrenia is associated with profound impairments in social cognition that result in reduced functioning. Among the constructs of social cognition, empathy refers to the ability to represent, infer, and share the feelings and emotions of others. Both theoretical accounts and neuroscience findings acknowledge the composite nature of empathy, making it difficult to operationalize. Acknowledging it is not correlated with empathic accuracy measures (1), self-reported empathy (i.e., questionnaires) has been regularly used to better understand how individuals are disposed to empathize with others. A recent meta-analysis focused on the widely used *Interpersonal Reactivity Index* (IRI) (2), which allows subscore decomposition, and showed that patients with schizophrenia exhibit reduced *empathic concern*, *perspective taking*, and *fantasy* and increased *personal distress* (3). The *Questionnaire of Cognitive and Affective Empathy* (QCAE) gathers relevant items from other tools, including the IRI, and provides measures of both *cognitive* (i.e., *perspective taking* and *online simulation*) and *affective* (i.e., *emotion contagion*, *proximal responsivity*, and *peripheral responsivity*) components (4). Psychometric evaluations of the English (4), Portuguese (5), Chinese (6), Italian (7), and French (8) versions of the QCAE in healthy populations suggest a five-factor structure. However, confirmatory factor analysis in patients with schizophrenia failed to replicate this finding and showed that only a two-factor structure achieved a correct fit after a comprehensive reorganization of item parcels (9).

Noting the paucity of information on the properties of social cognition instruments in clinical populations across different languages and cultures, we provide psychometric validation data for patients with schizophrenia spectrum disorders using the French version of the QCAE (**Supplementary Material 1**). Previous studies showed certain discrepancies in the factorial models (i.e., five-factor *vs.* bifactorial model), making it necessary to replicate the findings. Additional insights into the correlations with clinical and objective/subjective outcomes are also required to better understand the potential role of the self-reported empathy in schizophrenia assessment.

## Materials and Methods

We report data from adult volunteers with schizophrenia spectrum disorders (schizophrenia and schizoaffective disorder) referred to the Centers of Expertise for Schizophrenia (Foundation FondaMental) and included into the EVACO Study which is subordinated to the ongoing FACE-SZ Cohort (10). The local medical ethics committee (Comité de Protection des Personnes Ile-de-France XI, decision 2012-A00387-36) approved the study (EVACO, PHRC AOM11233).

The patients were diagnosed using a Structured Clinical Interview for assessing DSM-IV-R criteria. The study included only patients with clinically stable schizophrenia (no admission or treatment change in the past 4 weeks). Each participant received a complete description of the study in oral and written forms, gave written informed consent, and received monetary compensation.

Symptoms were assessed using the *Positive and Negative Symptom Scale* (11). Outcome measures, such as functioning and quality of life, were evaluated using the *Personal and Social Performance Scale* (PSP) (12) and the *Self rating Quality of Life Scale* (S-QoL) (13), respectively.

**Figure 1:**
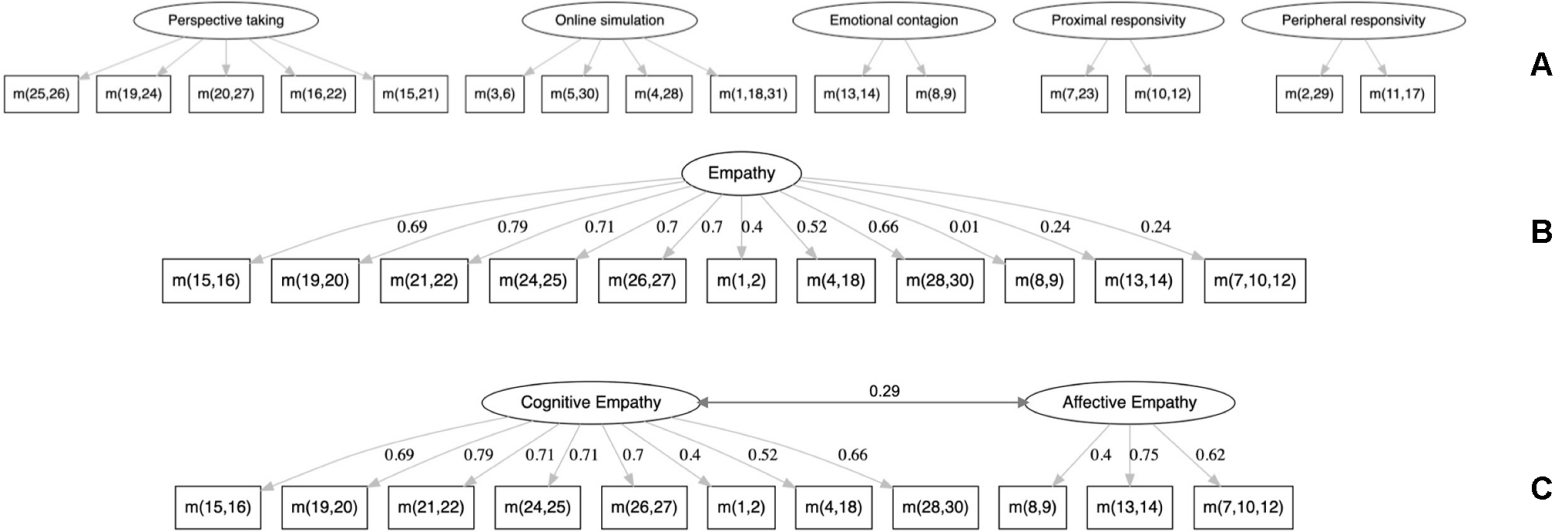
The three confirmatory factor analysis (CFA) models tested. **(A)** Five-factor first-order structure from (4). **(B)** Unique-factor first-order structure, without *peripheral responsivity* and parcellations, from (9). **(C)** Two-factor first-order structure from (9). *m*(*x,y,z*) signifies the mean of items *x*, *y*, and *z*.

QCAE items were parcelled following the various procedures proposed by Reniers *et al.* (2011) and Horan *et al.* (2015). Confirmatory factor analyses (CFA) were then conducted using the R Lavaan package (14) to compute the robust fit indices of three models (**Figure 1**): 1) a five-factor first-order structure (*perspective taking*, *online simulation*, *emotion contagion*, *proximal responsivity*, and *peripheral responsivity*) (4), 2) a unique-factor first-order model with the revised item parcellation proposed by (9), and 3) a two-factor first-order structure with *affective* and *cognitive* factors from the same revised parcellation. The retained model was tested for correlations with demographic and clinical variables (Bonferroni correction for multiple tests was applied).

## Results

### Demographic Data and Clinical Assessment

In total, 105 males and 28 females were recruited. Ninety-seven were diagnosed with schizophrenia and 36 with schizoaffective disorders. Their mean age was 31.6 ± 7.8 years. Their mean scores by the PANSS were 19.3 ± 7.6 for negative symptoms, 14.3 ± 5.5 for positive symptoms, and 33.4 ± 9.6 for general psychopathology.

### Confirmatory Factor Analysis

The subscores are reported in **Table 1**. The five-factor first-order model converged (RMSEA = 0.067, *p* of close fit = 0.088, 90% CI [0.046–0.087], CFI = 0.90, SRMR = 0.062). However, *peripheral responsivity* did not provide reliable estimates of regression coefficients with its two parcels. Metrological problems were previously reported with this subscore (9). Thus, we followed the recommendation of the authors to remove it and revised the parcel computation.

**Table 1:** Mean, standard deviations, and quantiles of the different scores with their formulae.

Score	Formula	Mean	SD	Q0.10	Q0.25	Q0.50	Q0.75	Q0.90
**Aff**	EC + ProxR + PeriR	27.44	4.7	21	24	21	30	33
EC	8 + 9 + 13 + 14	8.43	2.34	5	7	5	10	11
ProxR	7 + 10 + 12 + 23	8.85	2.3	6	7	6	11	11
PeriR	2 + 11 + 17 + 29	10.16	2.12	7	9	7	11	13
**Cog**	PT + OS	43.79	8.75	33	38	33	50	55
PT	15 + 16 + 19 + 20 + 21 + 22 + 24 + 25 + 26 + 27	23.76	5.54	16	20	16	27	31
OS	1 + 3 + 4 + 5 + 6 + 18 + 28 + 30 + 31	20.03	4.32	15	18	15	23	25
**AffRev**	ECRev + ProxRRev	15.08	3.63	10	13	10	17	19
ECRev	8 + 9 + 13 + 14	8.43	2.34	5	7	5	10	11
ProxRRev	7 + 10 + 12	6.65	1.95	4	5	4	8	9
**CogRev**	PTRev + OSRev	37.35	7.74	27^9^	32	27	43	47
PTRev	15 + 16 + 19 + 20 + 21 + 22 + 24 + 25 + 26 + 27	23.76	5.54	16	20	16	27	31
OSRev		13.59	3.12	10	12	10	15	18

Numbers indicate the Questionnaire of Cognitive and Affective Empathy (QCAE) item numbers. AffRev and CogRev indicate the revised scores following the parcel revision of Horan et al (9). Aff, affective empathy; EC, emotional contagion; ProxR, proximal responsivity; PeriR, peripheral responsivity; Cog, cognitive empathy; PT, perspective taking; OS, online simulation.

We thus tested the unique-factor model, which exhibited poor fit indices (RMSEA = 0.11, *p* of close fit = 0.000, 90% CI [0.091–0.137], CFI = 0.801, SRMR = 0.093).

The two-factor model adequately fits the data (RMSEA = 0.084, *p* of close fit = 0.019, 90% CI [0.058–0.109], CFI = 0.896, SRMR = 0.074), with the indices comparable to those reported by Horan *et al.* (9): RMSEA = 0.089, 90% CI [0.064–0.11], CFI = 0.91, SRMR = 0.069. All standardized factor loadings were above 0.3 and significant (*p* < 0.01). The correlation between the cognitive and affective factors was estimated to be 0.29 (*p* < 0.05). Note that this model gives similar results when applied to the subgroup of patients with schizophrenia (**Supplementary Table 1**).

### Association of Revised QCAE Scores With Demographic and Clinical Variables

Concerning direct comparisons, males scored higher than females at a trend level for the *affective* and *proximal responsivity* subscores (Welch tests: 15.4 *vs.* 14.0, *t* = 1.8, *df* = 45.4, *p* = 0.07 and 6.8 *vs.* 6.0, *t* = 1.9, *df* = 42.3, *p* = 0.06, respectively). Neither the age, the PANSS subscores nor the PSP exhibited significant corrected correlations with the QCAE subscores (**Supplementary Table 2**). The S-QoL total score correlated positively with the *emotional contagion* subscore (*r* = 0.29, *p* < 0.05 corrected) and showed a trend with the *affective* subscore (*r* = 0.26, *p* = 0.11 corrected). These findings are similar to those using the original subscores (4).

## Discussion

Questionnaires such as the QCAE provide simple means to assess subjective aspects of empathy through self-evaluation. The translation of this scale into French required the gathering of psychometric data for patients with schizophrenia spectrum disorders and further exploration of the main properties of this instrument. Our results confirm those of the study of Horan et al., showing that unless a better solution is proposed, the fit of the *affective* and *cognitive* bifactorial model, excluding the *peripheral responsivity* parcels, is acceptable and may be used for research. As our population was defined with broader criteria (adding schizoaffective disorders to the group) to be representative of the population with psychotic disorders, it is worth noting that the proposal of Horan et al. can still be used.

The retained model differed from that of studies in the healthy population, which favored a five-factor first-order structure (4–8). One interpretation is that healthy subjects may exert and assess their own social disposition in a more diverse way than patients with schizophrenia spectrum disorders who may poorly discriminate between them. A consequence for research is that caution should be used when comparing patients to healthy controls with this instrument as core structural properties depend on the group. Another consequence is that the *peripheral responsivity* subscore should be considered cautiously or ignored for patients with schizophrenia spectrum disorders as its items do not appear to cover a reliable construct.

Although this was only found at a trend level, self-reported proximal responsivity (i.e., emotional responsiveness to the moods of relatives) was found to be lower in females than males, and other subscores showed comparisons in the same direction. This was quite unexpected as the opposite effect was reported in healthy subjects by Reniers et al. (4). Contrary to Michaels et al., we did not find a significant correlation of empathy measures with functioning (9, 15). Similarly, psychopathology did not reveal significant relationships with empathy dispositions. Taken together with the small effect-sized findings of other studies (9, 15), these negative results emphasize the importance of investigating larger populations to determine the importance of the putative clinical expression of dispositional empathy.

In the absence of a relationship with functioning, the results with quality of life are of interest. *Affective empathy*/*emotional contagion* was positively associated with quality of life ratings, whereas *cognitive empathy*/*perspective taking* were negatively associated at a trend level. The items pertaining to the former dimension of empathy have in common that they describe the emotional disposition of the subjects toward others. The items of *emotional empathy* reflect a sense of closeness to others, which may explain the positive association with quality of life. In contrast, the negative correlation between *perspective taking* and quality of life is intriguing and should be investigated further because this did not reach the corrected significance threshold. However, the identification of factors that negatively influence quality of life is of clinical importance as they may favor depression and suicidality (16).

In conclusion, this study demonstrates the possibility of using self-administered empathy questionnaires in patients with schizophrenia spectrum disorders and suggests, through confirmatory factor analysis, that the structure of the QCAE tool is robust to language change in a clinical population. Intercultural studies should give priority to establishing the instrument’s properties in terms of measurement invariance in order to open up the possibility of making large-scale comparisons of international populations.

## Data Availability Statement

The datasets generated for this study will not be made publicly available. These data belong to the FondaMental network of Center of Expertise.

## Ethics Statement

The studies involving human participants were reviewed and approved by Comité de Protection des Personnes Ile-de-France XI, decision 2012-A00387-36. The patients/participants provided their written informed consent to participate in this study.

## Author Contributions

Conceptualization: EB-G, PR, and CP. Methodology: EB-G and PR. Formal analysis: EB-G, PR, and ME. Investigation: MU, BA, LB, DC, IC, CD, JD, GF, CL, SL, JM, DM, SP, PS, FS, HY, and AZ-B. Writing—preparation of the original draft: EB-G, PR, and ME. Writing—review and editing: all authors. Supervision and project administration: EB-G. Funding acquisition: EB-G and CP.

## Funding

The study was supported by the Centre Hospitalier de Versailles, Le Chesnay, France and the Fondation FondaMental, Créteil, France; and funded by the Programme Hospitalier de Recherche Clinique (AOM11233); the Investissements d’Avenir program managed by the Agence Nationale de la Recherche (ANR-11-IDEX-0004-02 and ANR-10-COHO-10-01); and the Institut National de la Santé et de la Recherche Médicale.

## Conflict of Interest

The authors declare that the research was conducted in the absence of any commercial or financial relationships that could be construed as a potential conflict of interest.
