# Supplementary material for: Confirmation of a Two-Factor Solution to the Questionnaire of Cognitive and Affective Empathy in a French Population of Patients With Schizophrenia Spectrum Disorders
Source: Front Psychiatry. 2019 Oct 25;10:751. doi: 10.3389/fpsyt.2019.00751 (PMC6823714; doi:10.3389/fpsyt.2019.00751)
Supplement: Supplementary file 1 [file Table_1.pdf]

Supplementary Table S1. CFA results of the Horan et al's two-factors model in whole population (schizophrenia and schizoaffective disorder) and in the sub-population with schizophrenia.

	Whole population	Schizophrenia
N	133	97
RMSEA	0.084	0.086
P of close fit	0.019	0.04
90% CI	[0.058 0.109]	[0.052 0.12]
CFI	0.896	0.88
SRMR	0.074	0.081
