# Supplementary material for: Confirmation of a Two-Factor Solution to the Questionnaire of Cognitive and Affective Empathy in a French Population of Patients With Schizophrenia Spectrum Disorders
Source: Front Psychiatry. 2019 Oct 25;10:751. doi: 10.3389/fpsyt.2019.00751 (PMC6823714; doi:10.3389/fpsyt.2019.00751)
Supplement: Supplementary file 2 [file Table_2.pdf]

Supplementary Table S2. Pearson's correlations of QCAE scores and subscores with clinical variables. The number of subjects with complete data are provided for each variable. * $p < 0.05$ with Bonferroni correction; \$ $p < 0.05$ without correction.

	AffRev	ECRev	ProxRRev	CogRev	PTRev	OSRev
Age (N = 133)	0.09	0.06	0.09	0.05	0.02	0.09
PANSS negative (N = 132)	-0.11	-0.14	-0.04	0.03	0.01	0.06
PANSS positive (N = 132)	-0.03	0.05	-0.11	-0.02	-0.01	-0.03
PANSS general (N = 131)	-0.18 $	-0.19 $	-0.11	0.09	0.07	0.09
PSP score (N = 125)	0.14	0.12	0.11	-0.11	-0.11	-0.08
S-QoL total (N = 127)	0.26 $	0.29 *	0.13	-0.19 $	-0.17 $	-0.16
