# Supplementary material for: Confirmation of a Two-Factor Solution to the Questionnaire of Cognitive and Affective Empathy in a French Population of Patients With Schizophrenia Spectrum Disorders
Source: Front Psychiatry. 2019 Oct 25;10:751. doi: 10.3389/fpsyt.2019.00751 (PMC6823714; doi:10.3389/fpsyt.2019.00751)
Supplement: Supplementary file 3 [file DataSheet_1.doc]

QCAE

Code sujet: Date:

QCAE / Versailles / EBG / PR

| Le gens éprouvent des sentiments différents selon les situations. Dans ce qui suit, plusieurs traits de caractère vous seront présentés qui pourront s’appliquer à vous à différents degrés. Lisez chacune de ces caractéristiques et indiquez à quel point vous êtes en accord ou en désaccord avec la proposition en cochant la case correspondante. Répondez rapidement et avec sincérité. | | Parfaitement d’accord | Assez d’accord | Pas vraiment d’accord | Pas d’accord du tout |
| --- | --- | --- | --- | --- | --- |
| 1. | Parfois, je trouve difficile de voir les choses du point de vue d’une autre personne. |  |  |  |  |
| 2. | D’habitude je garde mon objectivité quand je regarde un film ou quand je joue, et je ne me laisse pas souvent entrainer complètement dedans. |  |  |  |  |
| 3. | J’essaie de voir s’il y a des désaccords chez les autres avant de prendre une décision. |  |  |  |  |
| 4. | Des fois, j’essaie de mieux comprendre mes amis en imaginant les choses de leur point de vue. |  |  |  |  |
| 5. | Quand je suis peiné par quelqu’un, j’essaie d’habitude de me mettre à sa place pour un moment. |  |  |  |  |
| 6. | Avant de critiquer quelqu’un, j’essaie d’imaginer ce que je ressentirais si j’étais à sa place. |  |  |  |  |
| 7. | Je suis souvent impliqué(e) émotionnellement avec les problèmes de mes amis. |  |  |  |  |
| 8. | J’ai tendance à devenir nerveux(se) quand les autres autour de moi semblent être nerveux. |  |  |  |  |
| 9. | Les gens avec qui je suis ont une forte influence sur mon humeur. |  |  |  |  |
| 10. | Cela m’affecte beaucoup quand un de mes amis paraît avoir de la peine. |  |  |  |  |
| 11. | Je me sens souvent très impliqué(e) dans les sentiments d’un personnage de film, de théâtre ou de roman. |  |  |  |  |
| 12. | Je suis très contrarié(e) quand je vois quelqu’un pleurer. |  |  |  |  |
| 13. | Je suis heureux(se) quand je suis avec un groupe enjoué et triste quand les autres sont moroses. |  |  |  |  |
| 14. | Cela me soucie quand d’autres sont soucieux ou paniqués. |  |  |  |  |
| 15. | Je peux facilement dire si quelqu’un veut engager la conversation. |  |  |  |  |
| 16 | Je me rends compte rapidement si quelqu’un dit une chose mais veut en dire une autre. |  |  |  |  |
| 17. | Il est difficile pour moi de voir pourquoi certaines choses soucient autant les gens. |  |  |  |  |
| 18. | Je trouve qu’il m’est facile de me mettre à la place d’une autre personne. |  |  |  |  |
| 19. | Je sais bien prédire comment va se sentir une autre personne. |  |  |  |  |
| 20. | Je m’en rends compte rapidement quand quelqu’un dans un groupe se sent mal à l’aise ou gêné. |  |  |  |  |
| 21. | Les autres me disent que je sais bien comprendre ce qu’ils ressentent ou ce qu’ils pensent. |  |  |  |  |

| QCAE | | Parfaitement d’accord | Assez d’accord | Pas vraiment d’accord | Pas d’accord du tout |
| --- | --- | --- | --- | --- | --- |
| 22. | Je peux facilement dire si quelqu’un d’autre est intéressé ou ennuyé par ce que je raconte. |  |  |  |  |
| 23. | Les amis me parlent de leurs problèmes car ils disent que je suis très compréhensif(ve). |  |  |  |  |
| 24. | Je me rends compte quand je dérange même si l’autre personne ne me le dis pas. |  |  |  |  |
| 25. | J’arrive facilement à savoir de quoi une autre personne voudrait parler. |  |  |  |  |
| 26. | Je peux dire si quelqu’un masque ses vraies émotions. |  |  |  |  |
| 27. | Je sais bien prédire ce qu’une autre personne va faire. |  |  |  |  |
| 28. | Je sais généralement bien évaluer le point de vue d’une autre personne, même si je ne suis pas d’accord avec. |  |  |  |  |
| 29. | Je suis habituellement détaché(e) émotionnellement quand je regarde un film. |  |  |  |  |
| 30. | Je prends toujours en considération les sentiments des autres avant de faire quelque chose. |  |  |  |  |
| 31. | Avant de faire quelque chose j’essaie de voir comment mes amis vont réagir. |  |  |  |  |
